# Three newly established immortalized mesothelial cell lines exhibit morphological phenotypes corresponding to malignant mesothelioma epithelioid, intermediate, and sarcomatoid types, respectively

**DOI:** 10.1186/s12935-021-02248-5

**Published:** 2021-10-18

**Authors:** Tatsuhiro Sato, Hayao Nakanishi, Ken Akao, Maho Okuda, Satomi Mukai, Tohru Kiyono, Yoshitaka Sekido

**Affiliations:** 1 Division of Cancer Biology, Aichi Cancer Center Research Institute, 1-1 Kanokoden, Chikusa-ku, Nagoya, Aichi 464-8681 Japan; 2 Department of Gastroenterological Surgery, Nagoya University Graduate School of Medicine, 65 Tsurumai-cho, Showa-ku, Nagoya, Aichi 466-8550 Japan; 3 Institute for Environmental and Gender-Specific Medicine, Juntendo University Graduate School of Medicine, Urayasu, Chiba 279-0021 Japan; 4 Project for Prevention of HPV-Related Cancer, Exploratory Oncology Research and Clinical Trial Center, National Cancer Center, Kashiwanoha 6-5-1, Kashiwa City, Chiba 277-8577 Japan; 5 Division of Molecular and Cellular Oncology, Nagoya University Graduate School of Medicine, 65, Tsurumai-cho, Showa-ku, Nagoya, Aichi Japan

**Keywords:** Mesothelioma, Mesothelial cell, EMT, TGF-β, IL-1β

## Abstract

**Background:**

Malignant mesothelioma (MM) is a very aggressive tumor that develops from mesothelial cells, mainly due to asbestos exposure. MM is categorized into three major histological subtypes: epithelioid, sarcomatoid, and biphasic, with the biphasic subtype containing both epithelioid and sarcomatoid components. Patients with sarcomatoid mesothelioma usually show a poorer prognosis than those with epithelioid mesothelioma, but it is not clear how these morphological phenotypes are determined or changed during the oncogenic transformation of mesothelial cells.

**Methods:**

We introduced the E6 and E7 genes of human papillomavirus type 16 and human telomerase reverse transcriptase gene in human peritoneal mesothelial cells and established three morphologically different types of immortalized mesothelial cell lines.

**Results:**

HOMC-B1 cells exhibited epithelioid morphology, HOMC-A4 cells were fibroblast-like, spindle-shaped, and HOMC-D4 cells had an intermediate morphology, indicating that these three cell lines closely mimicked the histological subtypes of MM. Gene expression profiling revealed increased expression of NOD-like receptor signaling-related genes in HOMC-A4 cells. Notably, the combination treatment of HOMC-D4 cells with TGF-β and IL-1β induced a morphological change from intermediate to sarcomatoid morphology.

**Conclusions:**

Our established cell lines are useful for elucidating the fundamental mechanisms of mesothelial cell transformation and mesothelial-to-mesenchymal transition.

**Supplementary Information:**

The online version contains supplementary material available at 10.1186/s12935-021-02248-5.

## Background

The mesothelium is a membrane covering the body’s serous cavities and internal organs, where mesothelial cells form a monolayer of specialized pavement-like cells on both surfaces [1]. Mesothelial cells have characteristic microvilli at the cell surface, which are thought to play a role in protecting the mesothelium from damage caused by surface friction with organ movement. In addition, mesothelial cells show other important characteristics, including phagocytosis, anoikis resistance, and epithelial-to-mesenchymal transition (EMT), which has been termed the mesothelial-to-mesenchymal transition (MMT) [1, 2].

Malignant mesothelioma (MM) develops from mesothelial cells; however, the exact mechanism of this process has not yet been clearly determined. MM occurs in the pleura, pericardium, peritoneum, and tunica vaginalis. MM is caused by asbestos, and clinically overt MM tumors are usually diagnosed after a long latency of ~30–40 years [3, 4]. The number of patients with MM is increasing due to the large amounts of asbestos used in the past [5]. Clinically, curative treatment modalities are still limited for patients with MM, and the prognosis of patients is usually poor [6, 7].

The main genomic abnormalities in MM are frequent inactivating mutations in several key tumor suppressor genes, including *CDKN2A*, *NF2*, and *BAP1* [8, 9]. *NF2* encodes the Merlin protein, which regulates the Hippo signaling pathway. The Hippo pathway controls cell growth and organ size by regulating the transcriptional coactivators of YAP1 and TAZ [10, 11]. Once the Hippo pathway is suppressed, activated YAP1 and TAZ bind to TEAD transcription factors and enhance the transcription of pro-oncogenic genes, including connective tissue growth factor (*CTGF*). Meanwhile, TGF-β stimuli to mesothelial cells can activate SMAD proteins, and the activated SMAD4 can form a protein complex with YAP1/TEAD, which further enhances the transcription of downstream genes. Thus, a strong crosstalk between the TGF-β and Hippo pathways exists in MM cells, in which both TGF-β signaling activation and Hippo pathway suppression upregulate CTGF expression to induce tumor metastasis and EMT [12, 13].

MM is classified into three major histological subtypes: epithelioid, biphasic, and sarcomatoid. Notably, sarcomatoid MM usually shows worse malignant behavior than epithelioid MM. Thus, much interest has been paid to the cause of the differences in histological subtypes during MM development. In this regard, MM with epithelioid type shows more frequent *BAP1* mutations, while MM with non-epithelioid type has more frequent *NF2*, *LATS2*, and *TERT* promoter mutations [1, 8, 14]. Although several differences in genomic alterations have been observed between epithelioid and sarcomatoid types, the exact mechanisms responsible for the different histological subtypes of MM cells remain unidentified.

Immortalized mesothelial cells are essential and useful control cells for a variety of experiments using MM cells. However, few immortalized mesothelial cell lines are available globally. After searching five well-known cell banks, namely, the American Type Culture Collection (Manassas, VA, USA), the Coriell Cell Repository (Camden, NJ, USA), European Collection of Cell Cultures (London, UK), Japanese Collection of Research Bioresources Cell Bank (Osaka, Japan), and RIKEN BioResource Research Center (Ibaraki, Japan), we found only two immortalized mesothelial cell lines (MeT-5A and LP-9) that have been registered and available. In this study, we established three immortalized mesothelial cell lines with distinct morphologies from a patient and analyzed their differentiation potential.

## Materials and methods

### Sample collection

Primary mesothelial cells were obtained from the greater omentum of a 51-year-old Japanese male patient who had undergone surgery for early gastric cancer at Aichi Cancer Center Hospital. This study was approved by the Ethics Committee at the Aichi Cancer Center (Nagoya, Aichi, Japan), and written informed consent was obtained from all patients.

### Establishment of HOMC cell lines

Small pieces of the greater omentum were treated with dispase, and mesothelial cells with epithelial-like morphology were separated by mechanical scraping. Primary mesothelial cells were retrovirally transduced with human papillomavirus 16 E6/E7 (pCLXN-16E6E7) and human TERT (pCMSCVpuro-hTERT) [15], selected with 200 μg/mL G418 and 1 μg/mL puromycin, and subcloned using the ring cloning method. The cell lines used in the comparative experiments, MeT-5A and NCI-H2052, were purchased from the American Type Culture Collection (Rockville, MD, USA), and NCI-H2452 was a gift from Dr. Adi F. Gazdar. The NCCIT cell line was purchased from the Japanese Collection of Research Bioresources Cell Bank. All cell lines were cultured in RPMI-1640 medium supplemented with 10% fetal bovine serum (FBS) and 1% antimicrobial at 37 °C in a humidified incubator with 5% CO_2_. The cell proliferation rate was measured based on cell confluency, which was calculated using the IncuCyte S3 Live-Cell Analysis System (Essen Biosciences, Ann Arbor, MI, USA).

### Antibodies

Antibodies against NF2 (#12888), p16 (#92803), MET (#8198), phospho-MET (#3133), Oct-4A (#2840), Sox2 (#3579), Nanog (#4903), and KLF4 (#4038) were obtained from Cell Signaling Technologies (Danvers, MA, USA). Anti-BAP1 was purchased from Santa Cruz Biotechnology (#sc-28383, Santa Cruz, CA, USA), anti-acetylated tubulin from Sigma (#T7451, St. Louis, MO, USA), and anti-actin from Abcam (#ab14128, Cambridge, UK).

### Scanning electron microscopy

Cells plated on a 15-mm coverglass were fixed with 2.5% glutaraldehyde in phosphate-buffered saline (PBS) (pH 7.4) overnight at 4 °C and then incubated in 1% osmium tetroxide for 1 h. The cells were dehydrated in a series of increasing concentrations (50–100%) of ethanol and then dried in absolute ethanol using the critical point drying method. This sample was observed and photographed using a scanning electron microscope (SEM, JSM-7610F; JEOL).

### Immunocytochemistry

Samples were fixed in PBS containing 4% paraformaldehyde (PFA) at room temperature for 15 min, followed by PBS containing 0.1% TritonX-100 (PBS-T) for 15 min. Next, the samples were incubated with a specific antibody solution (1:200 dilution) in PBS-T containing 1% bovine serum albumin (#10735086001; Sigma) at 4 °C overnight. After washing with PBS-T, the samples were incubated with 66 nM Alexa Fluor 546-conjugated phalloidin (#A22283; Thermo Fisher Scientific, Waltham, MA) and Alexa Fluor 488-conjugated secondary antibody solution (#A21202; Thermo Fisher Scientific, 1:200 dilution) in PBS-T containing 1% bovine serum albumin for 1 h at room temperature, washed with PBS-T, and mounted in Vectashield mounting medium containing DAPI (Vector Laboratories). Fluorescence images were obtained using a Carl Zeiss LSM800 confocal laser scanning microscope (Oberkochen, Germany).

### Quantitative RT-PCR

Total RNA was prepared using an RNeasy Plus RNA extraction kit (Qiagen, Tokyo, Japan) according to the manufacturer’s protocol. Random-primed, first-strand cDNA was synthesized from total RNA using the ReverTra Ace qPCR RT Kit (Toyobo, Osaka, Japan). qRT-PCR analyses were performed in triplicate using the KAPA SYBR Fast qPCR kit (KAPA Biosystems, Boston, MA, USA) in the QuantStudio 3 system (Applied Biosystems, Foster City, CA, USA). Relative gene expression normalized to the expression of β-actin as a reference gene was determined using the relative standard curve method. The primer sequences used in this study were as follows: β-*Actin*, 5′-*CCAACCGCGAGAAGATGA*-3′ and 5′-*CCAGAGGCGTACAGGGATAG*-3′; *HPV-16E7*, 5′-*CAACTGATCTCTACTGTTATGAGCAA*-3′ and 5′-*CCAGCTGGACCATCTATTTCA*-3′; *HPV-16E6*, 5′-*GTCATATACCTCACGTCGCAG*-3′ and 5′-*AGCGACCCAGAAAGTTACCAC*-3′; *hTERT*, 5′-*GCCTTCAAGAGCCACGTC*-3′ and 5′-*CCACGAACTGTCGCATGT*-3′; *Calretinin*, 5′-*GATCCTGCCAACCGAAGAGAAC*-3′ and 5′-*CGATGTAGCCACTCCTGTCTGT*-3′; *Mesothelin*, 5′-*ACGTGGGGTCCCAGAAGT*-3′ and 5′-*GAATGTGAGCATGGACTTGG*-3′; *WT-1*, 5′-*CGAGAGCGATAACCACACAACG*-3′ and 5′-*GTCTCAGATGCCGACCGTACAA*-3′;

*E-cadherin,* 5-*GCTGGCTCAAGTCAAAGTCC*-3 and 5-*CCCGGGACAACGTTTATTAC*-3; *podoplanin*, 5-*GCGTAACCCTTCAGCTCTTTAG*-3 and 5-*TGGGGTCTTACTAGCCATCG*-3; *vimentin*, 5-*AGCCTCAGAGAGGTCAGCAA*-3 and 5-*AAAGTGTGGCTGCCAAGAAC*-3; *CTGF*, 5-*TGGAGATTTTGGGAGTACGG*-3 and 5-*CCTGCAGGCTAGAGAAGCAG*-3; *Snail*, 5-*TGCCCTCAAGATGCACATCCGA*-3 and 5-*GGGACAGGAGAAGGGCTTCTC*-3′.

### Phospho-RTK array analysis

The phosphorylation and activation of receptor tyrosine kinases (RTKs) were analyzed using the Human Phospho-RTK Array Kit (R&D Systems, Minneapolis, MN, USA) according to the manufacturer’s instructions. Briefly, cells were cultured in 10 cm plates in RPMI1640 without serum for 24 h and lysed using a lysis buffer (1% NP-40, 20 mM Tris–HCl, pH 8.0, 137 mM NaCl, 10% glycerol, and 1 × protease inhibitor cocktail [Sigma]). The arrays were blocked in blocking buffer and incubated with 500 μg of cell lysate overnight at 4 °C. The arrays were washed and incubated with a horseradish peroxidase-conjugated phospho-tyrosine detection antibody, treated with ECL, and exposed to a film. The intensity of each spot detected using a phospho-RTK array was measured using ImageJ software (NIH, Bethesda, MD, USA). The background intensity was determined and subtracted from each average signal.

### Western blot analysis

Cells were lysed with 1 × SDS sample buffer (3% SDS, 5% glycerol, 62 mM Tris–HCl [pH 6.8], 5% 2-mercaptoethanol) and incubated at 95 °C for 5 min. The proteins were resolved by 5–12% gradient polyacrylamide gel (#192–15201; Fujifilm, Osaka, Japan), transferred onto a PVDF membrane (#IPVH00010; Merck, Darmstadt, Germany) and probed with a 1000-fold dilution of antibodies and horseradish peroxidase (HRP)-conjugated secondary antibodies (Cell Signaling Technologies) in 5% skim milk. Protein bands were detected using ECL Prime Detection Reagent (#RPN2236; GE Healthcare, Chicago, IL, USA) and Amersham Imager 680 (GE Healthcare).

### Microarray analysis

Gene expression analysis was performed using the SurePrint G3 Human GE 8 × 60 K V2 Kit (#G4851B; Agilent Technologies, Palo Alto, CA, USA), as described previously [16]. Microarrays were scanned and converted into datasets using Agilent Micro Array Scanner and Feature Extraction software version 9.1. Flagged spots were treated as missing values. Probes treated as missing values in ˃50% of the samples were excluded from further analysis. Raw data were normalized by log2 conversion and *z*-score calculation [(individual log-changed signal intensity (LS) – mean score of all LS)/SD of all LS]. Normalized data were used for statistical analyses. GSEA (http://www.broad.mit.edu/gsea) [17] was used to evaluate the microarray dataset against a collection of gene sets in the Kyoto Encyclopedia of Genes and Genomes (KEGG) pathway database.

### Cytogenetic analysis

Chromosome preparations and GTG-banding were performed as previously described [18]. A total of 50 metaphases were karyotyped.

### Short tandem repeat (STR) and SNP analyses

Genomic DNA from HOMC cell lines was isolated using the PureLink Genomic DNA Mini Kit (Thermo Fisher Scientific). Fifteen STR sites (*D3S1358*, *TH01*, *D21S11*, *D18S51*, *Penta E*, *D5S818*, *D13S317*, *D7S820*, *D16S539*, *CSF1PO*, *Penta D*, *vWA*, *D8S1179*, *TPOX*, and *FGA*) and amelogenin were analyzed using PowerPlex 16 HS System (Promega, Madison, WI) and GeneMapper ID v3.2 Software (Thermo Fisher Scientific). Fifty-two SNP array analyses were performed identical.

### Tumorigenicity in nude mice

For mice, 15–20 g 6-week-old KSN (nu/nu) female nude mice were obtained from Japan SLC (Shizuoka, Japan). The mice were maintained in a controlled environment with 12-h light–dark cycle, room temperature of 22–24 °C, and humidity (55 ± 5%) and were given ad libitum access to food and water. The cultured cells (6–8 × 10^6^) of the passage numbers between 30 and 35 were washed, resuspended in PBS (0.2 mL), and injected subcutaneously into the left flank of mice within 1–2 weeks after obtaining. As a control, 0.2 mL of PBS alone was similarly injected into the right flank of the nude mice. The animals were examined weekly for tumor development and euthanized by cervical dislocation at the end of the study. All animal care was performed in accordance with the institutional guidelines stipulated by the Animal Experiment Committee of the Aichi Cancer Center Research Institute (Nagoya, Aichi, Japan).

### Statistical methods

Differences between two groups were statistically analyzed using the Student’s t-test. The *P*-values of all statistical tests were two-sided, and differences were considered significant at *P *< 0.05. qRT-PCR and cell proliferation assays were repeated at least thrice.

## Results

### Establishment of immortalized mesothelial cell lines

**Fig. 1:**
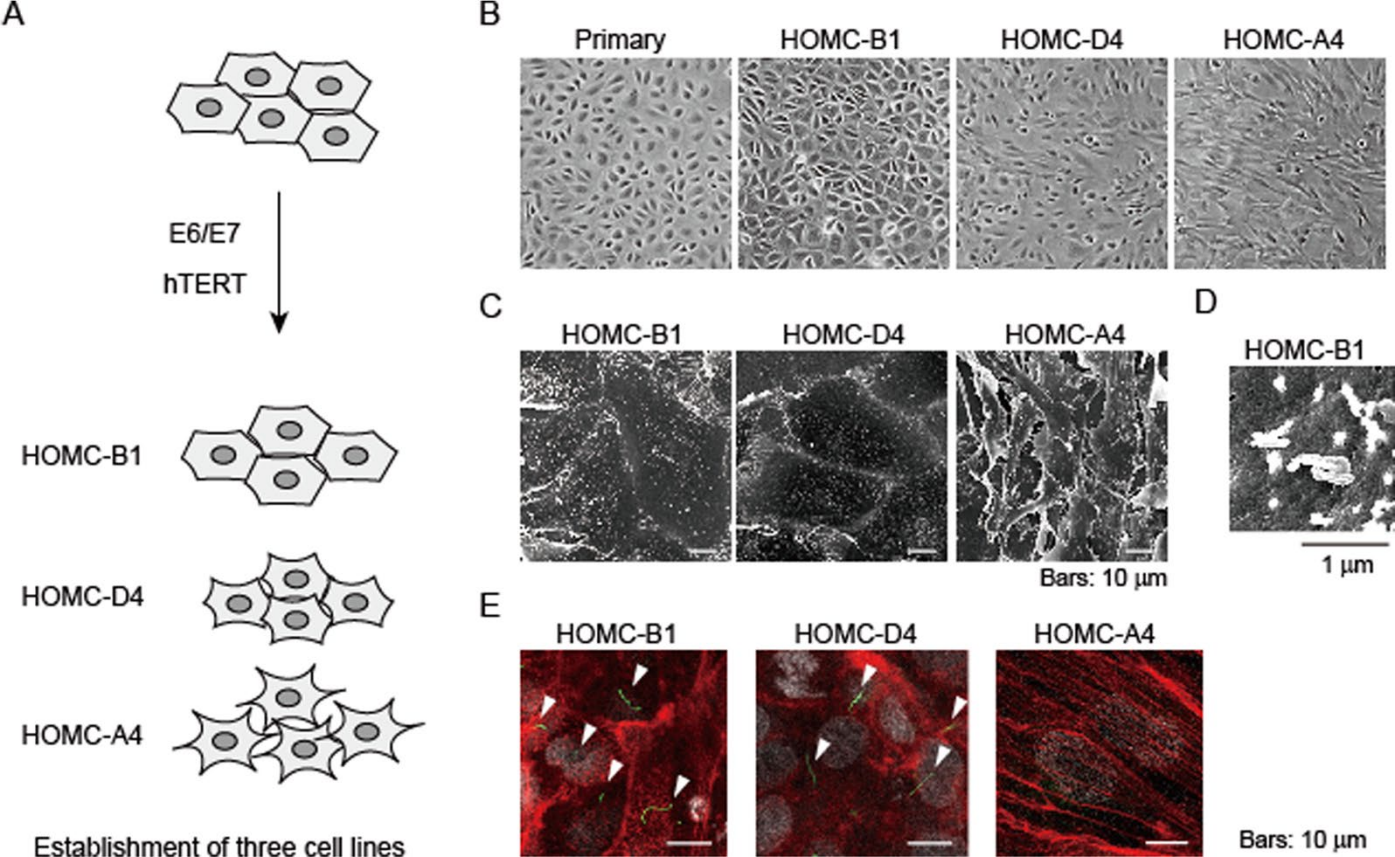
Establishment of three immortalized mesothelial cell lines. **A** Schematic procedure used for establishing HOMC cell lines. **B**–**D** Distinct cell morphologies of HOMC cells. Cells are observed using a light microscope (**B**) or with a scanning electron microscope (**C**, **D**), and representative photographs are shown. **E** HOMC cells form primary cilia. The cells are stained for F-actin (red), acetylated tubulin (green), and with DAPI (white) and observed using a confocal laser microscope. Representative immunofluorescence images are shown. Primary cilia are indicated by arrow heads

To establish immortalized mesothelial cell lines, we first obtained mesothelial cells from the omental tissue, which was resected from a patient with gastric cancer and cultured in RPMI-1640 medium supplemented with 10% FBS. Since human primary cells eventually undergo cell cycle arrest after a limited number of cell divisions, we introduced human papillomavirus 16 (*HPV16*) *E6* and E7 genes and human telomerase reverse transcriptase (*hTERT*) genes into the cells using retroviral vectors to ensure their continuous growth (Fig. 1A). Cells expressing these genes were selected using antibiotic treatment, and single-cell clones were separated using cloning rings. We successfully obtained three immortalized human omental mesothelial cell lines (hereafter abbreviated as HOMCs) with distinct cell morphologies (Fig. 1A).

HOMC-B1 cells exhibited a cobblestone-like epithelioid morphology similar to that of the parental primary mesothelial cells; HOMC-A4 cells displayed an elongated fibroblast-like morphology, and HOMC-D4 cells showed an intermediate cell shape (Fig. 1B). Scanning electron microscopic analyses detected numerous microvilli with a length of ~0.5–1 μm at the surface of HOMC-B1 and D4 cells (Fig. 1C, D). Immunocytochemical analysis detected primary cilia at the cell surface, which had a characteristic appearance of microtubule-based protrusions from the plasma membranes and are known to form during the growth quiescence phase of normal cells [19] (Fig. 1E). In contrast, we observed that HOMC-A4 cells showed fewer microvilli with insufficient development of primary cilia and that their cell edges overlapped with adjacent cell edges, consistent with fibroblastic morphology (Fig. 1C, E).

### Confirmation of cell identities, integrities, and mesothelial lineage

**Fig. 2:**
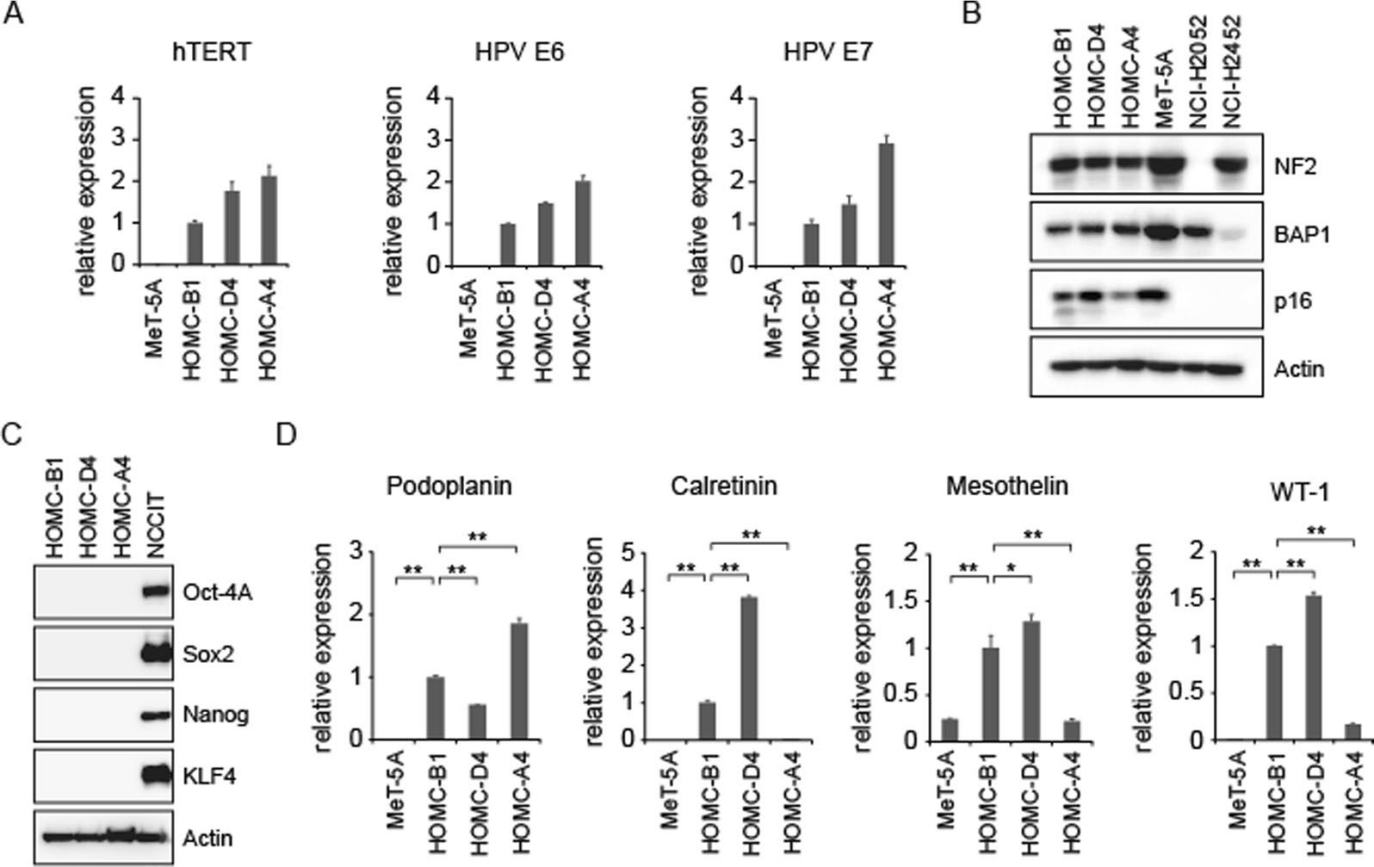
Expression of introduced genes, tumor suppressor proteins, and mesothelial markers. **A–D** Relative expression levels of indicated genes and proteins are analyzed using qRT-PCR (**A**, **D**) or western blotting (**B**, **C**). Two MM cell lines used as controls in **B** are NCI-H2052 cells with *CDKN2A* and *NF2* alterations and NCI-H2452 cells with *CDKN2A* and *BAP1* alterations. NCCIT teratoma cells in **C** were used as a control to express stem cell-related proteins. qRT-PCR was performed three times. Data in **A** and **D** represent the mean ± S.D. *P < 0.05; **P < 0.01; n.s., not significant.

The fact that we were able to establish three different phenotypic cell lines suggests that this may be because mesothelial cells are susceptible to induction of EMT and that these cell lines may be very useful for future studies. To confirm the identities of these cell lines from one patient, we performed STR and SNP array analyses and found that they had almost identical microsatellite and SNP patterns (Table 1). We next studied the expression levels of *hTERT*, *HPV E6*, and *E7* and found that each HOMC expressed comparable mRNA levels of the infected genes (Fig. 2A), suggesting that all exogenously induced genes functioned to maintain cell growth for each HOMC.

**Table 1:** Short tandem repeats profiles in HOMC cell lines

Locus	D3S1358	TH01	D21S11	D18S51	Penta E	D5S818	D13S317	D7S820
HOMC-B1	16, 18	6, 9	29, 30	13, 16	12, 19	10, 11	12	11, 12
HOMC-D4	16, 18	6, 9	29, 30	13, 16	12, 19	10, 11	12	11, 12
HOMC-A4	16, 18	6, 9	29, 30	13, 16	12, 19	10, 11	10, 12	11, 12

Locus	D16S539	CSF1PO	Penta D	Amelogenin	vWA	D8S1179	TPOX	FGA
HOMC-B1	9, 12	11, 12	10	x, y	14	13, 15	8, 9	21, 22
HOMC-D4	9, 12	11, 12	10	x, y	14	13, 15	8, 9	21, 22
HOMC-A4	9, 12	11, 12	10	x, y	14	13, 15	8, 9	21, 22

To assess the genomic stability of HOMC cell lines, we analyzed their chromosome numbers and karyotypes (Table 2). While certain changes in chromosomal number and structure were detected in all cell lines, the levels of abnormalities seemed to be much milder than those in MM cells [20]. This suggested that artificial chromosomal alterations were induced during the establishment of these lines and that HOMC cells had low-level chromosomal instability, probably due to functional p53 inactivation by HPV E6/E7 proteins.

**Table 2:** Karyotype analysis of HOMC cell lines

Cell line	Chromosome count							Total	Karyotype
	42	43	44	45	46	47	48		
HOMC-B1	1	7	19	21	2	0	0	50	80% 45, XY, − 13
HOMC-D4	0	3	10	24	10	0	0	47	55% 45, XY, − 13, − 22, + mar
HOMC-A4	0	0	1	6	38	1	0	46	45% 46, XY, − 14, − 20, + 2mar

Since chromosomal alterations were detected, we suspected that HOMC cells might have artificially lost the expression of key MM tumor suppressor proteins during establishment to acquire immortalization capacity. To exclude this possibility, we studied the expression of merlin, BAP1, and p16^Ink4a^ proteins, which are the three major tumor suppressors in MM cells. Compared to the two MM cell lines, NCI-H2052 and NCI-H2452, three HOMC cell lines, as well as MeT-5A immortalized mesothelial cells, expressed these tumor suppressor gene products (Fig. 2B). These results suggest that, despite chromosomal alterations, our established immortalized mesothelial cell lines maintain the genomic integrity of key genes. We further examined stem cell-related protein expression levels, but found no expression of Oct-4A, Sox2, Nanog, or KLF4 in any HOMC cell line, except for the teratoma cell line NCCIT (Fig. 2C).

Since intermediate- and sarcomatoid-type HOMCs showed very different cell morphologies from mesothelial cells, we also suspected that these cells might have lost mesothelial lineage phenotypes. To determine whether HOMCs expressed mesothelial lineage markers, we studied four well-known mesothelial cell markers, podoplanin, calretinin, mesothelin, and WT-1. We detected the expression of all these genes in HOMC -D4 and HOMC-B1 cell lines, indicating that they maintained the mesothelial lineage (Fig. 2C). Although HOMC-A4 cells also maintained the expression of calretinin, mesothelin, and WT1, they were much weaker than those in the other cells, suggesting that they might reflect the process of EMT.

### Differences in cell proliferation and cell growth signaling of HOMCs

**Fig. 3:**
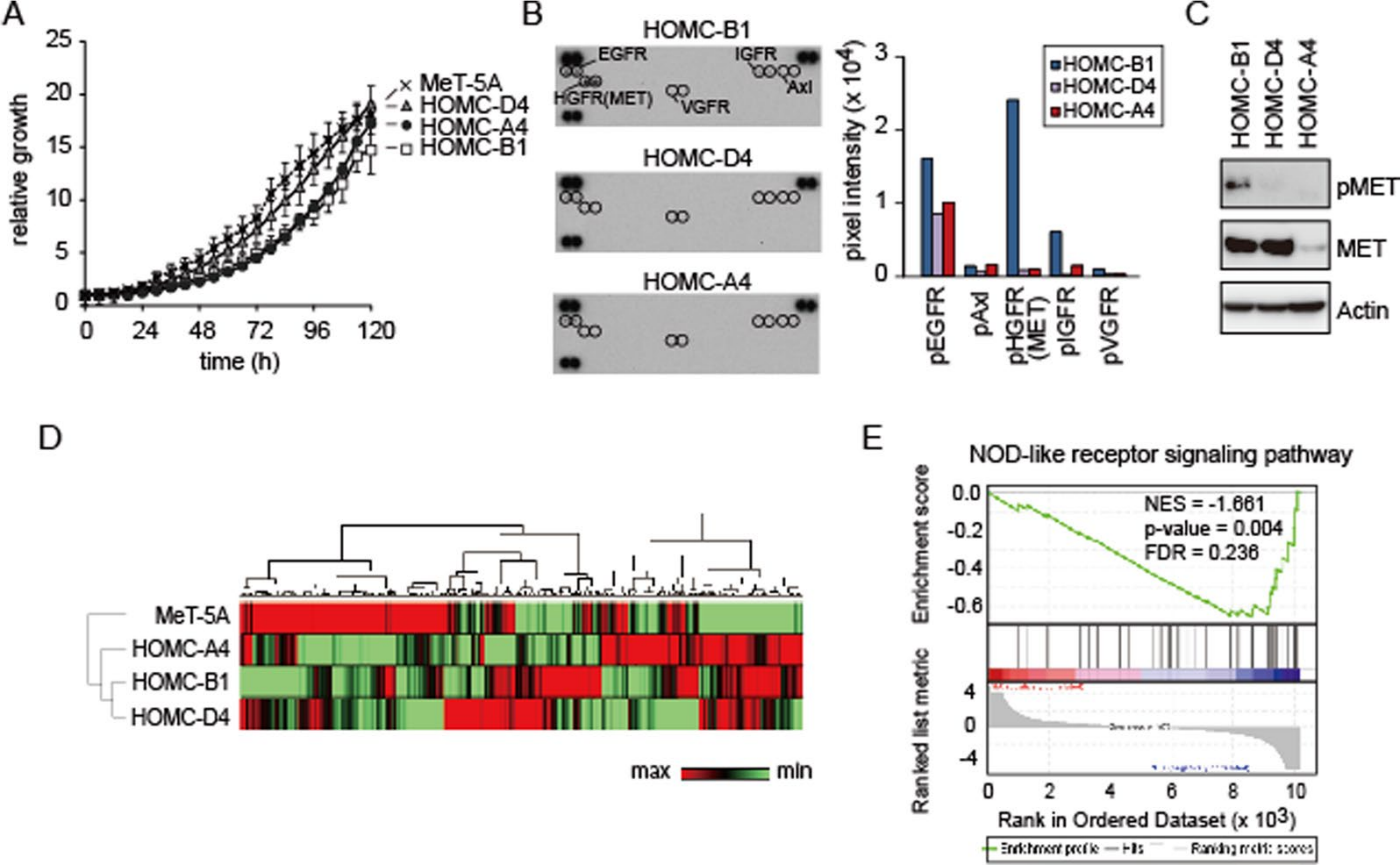
Receptor tyrosine kinase activation and gene expression profiles in HOMC cell lines. **A** Cell proliferation assay. The proliferation of each cell line is measured every 6 h using Incyte S3. Experiments of cell proliferation assay were repeated three times. **B** The phosphorylation of receptor tyrosine kinases (RTKs). Each HOMC cell line cultured under serum-starved conditions for 24 h is subjected to phospho-RTK array. Positive duplicate spots for individual RTKs are detected along the array. Three sets of two dots at the top corners and the bottom left represent positive controls and eight dots at the bottom right represent negative controls. Positive spots are quantified using ImageJ software and the results are shown on the right side. **C** Western blot analysis of MET and phospho-MET using HOMC cell lines. **D** Heatmap visualization of microarray data. Red or green colors indicate up- or down-regulated genes, respectively. The dendrogram is the results of hierarchical clustering using the average linkage method. **E** Gene set enrichment analysis (GSEA) showing enrichment of NOD-like receptor signaling pathway comparing between HOMC-A4 and HOMC-D4 cells. Significance was determined from the nominal *P*-values (*P*-value ≤ 0.01) and false discovery rate (FDR ≤ 0.25) obtained by GSEA. *NES* normalized enrichment score

Next, to determine whether morphological differences may affect cell growth, we compared the proliferation rates of the three HOMC cell lines and the MeT-5A cell line. We found that the HOMC-D4 line showed almost identical proliferation to the MeT-5A line, while the other two lines had slightly slower proliferation rates (Fig. 3A). Furthermore, since activation of the receptor tyrosine kinases (RTKs) is important for the proliferation of MM cells, we further investigated the phosphorylation status of RTKs. Previously, we examined the phosphorylation status of 42 RTKs in 15 MM and MeT-5A cell lines under serum-free culture conditions and reported that an average of 6.2 RTKs were phosphorylated, especially EGFR family members and MET [21]. We performed a phospho-RTK array to determine if there were differences in the patterns of RTK activation states in HOMC cell lines (Fig. 3B). HOMC cells showed a similar pattern of RTK activation to the MM and MeT-5A cell lines we previously reported [21], but a closer look revealed that EGFR was moderately (or slightly) phosphorylated in all three lines, while the phosphorylation levels of other common RTKs, such as MET, differed greatly among the cell lines (Fig. 3B, C). These results suggest that activation of several characteristic RTKs, including EGFR, is important for HOMC cell proliferation, as is the case with MM and MeT-5A cell lines, and that stimulation of exogenous factors may also be important in the tissue microenvironment, as suggested by the hypophosphorylation state of MET found in HOMC-D4.

### Gene expression profiles in HOMC cell lines

To determine the genes responsible for the differences in the cell morphology of the HOMC lines, we performed microarray analysis. Hierarchical clustering analysis of gene expression revealed that the three HOMC cell lines displayed different profiles from those of the MeT-5A cell line (Fig. 3D). Among the three HOMC lines, HOMC-A4 cells had a relatively different gene expression profile compared to the other two HOMC lines. We further performed gene set enrichment analysis (GSEA) for the expression data using the KEGG gene set file. Interestingly, the expression of genes associated with the NOD-like receptor signaling pathway was significantly different between the HOMC-A4 and HOMC-D4 lines (Fig. 3E). IL-1β, a member of the NOD-like receptor signaling pathway, is known to contribute to malignant transformation of malignant mesothelioma [22], suggesting that it may be involved in the formation of the fibroblast-like morphology of HOMC-A4.

**Fig. 4:**
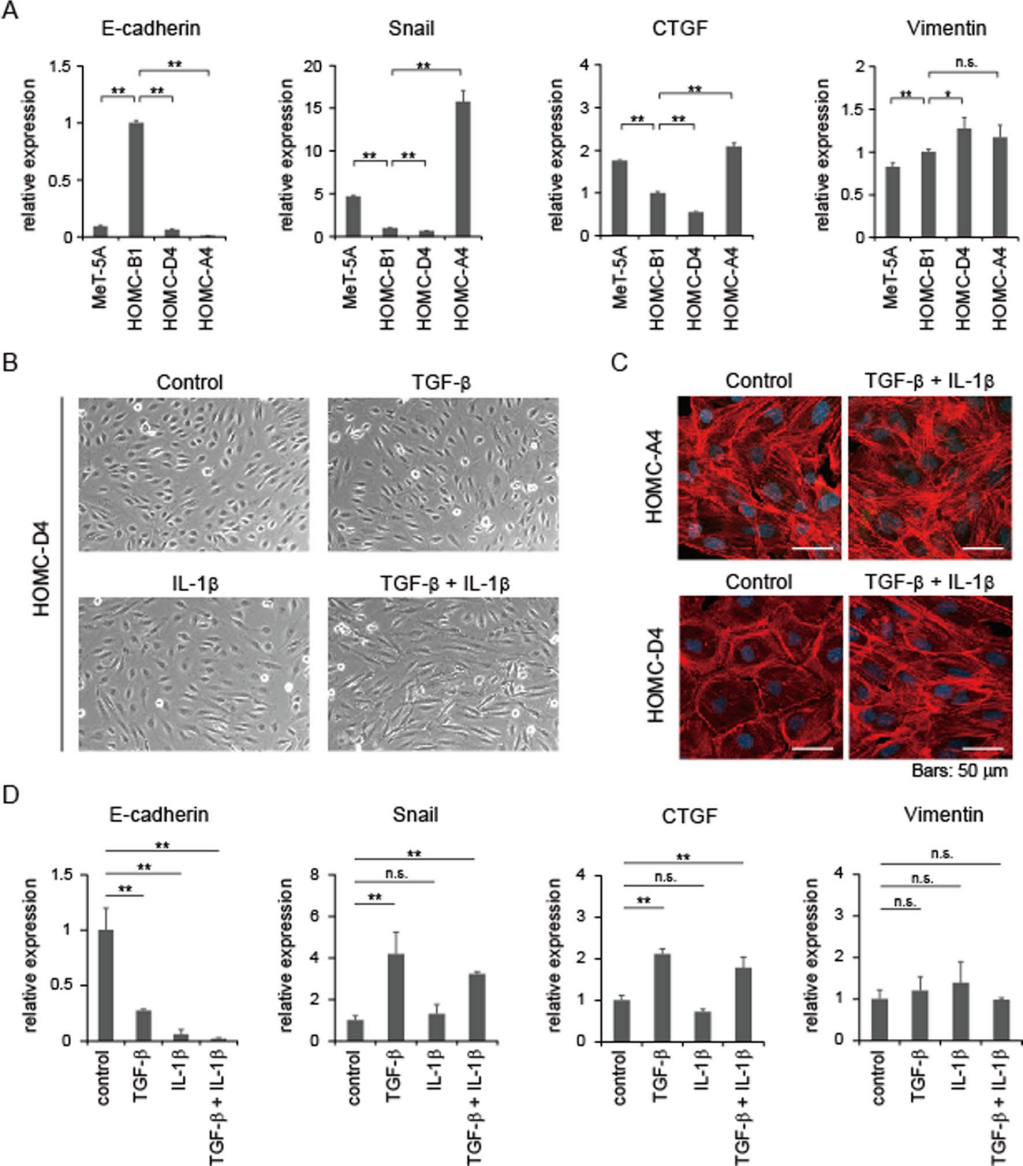
EMT-like morphological change in HOMC-D4 cells induced by TGF-β and IL-1β. **A** qRT-PCR analysis of EMT markers in HOMCs. **B**, **C** EMT-like change in HOMC-D4 cells in response to TGF-β and IL-1β treatment. Cells are treated with 2 ng/mL each of TGF-β and IL-1β individually or in combination for 24 h. Cell morphologies are observed under a light microscope (**B**) or a confocal laser microscope followed by immunostaining for F-actin (Red) and with DAPI (Blue) (**C**). Representative images are shown. **D** Altered gene expression of EMT markers in HOMC-D4 cells treated with TGF-β and IL-1β. After HOMC-D4 cells were administered with 2 ng/mL each of TGF-β and IL-1β individually or in combination for 24 h, the cells were lysed and subjected to qRT-PCR analysis. qRT-PCR was performed three times. Data represent the mean ± S.D. *P < 0.05; **P < 0.01; n.s., not significant.

To further investigate the differences in the morphologies of HOMC cell lines, we measured the expression levels of genes associated with EMT using quantitative real-time PCR (Fig. 4A). The expression level of E-cadherin, an epithelial marker, was high in HOMC-B1 cells, but low in the other two HOMCs and MeT-5A cells. Two EMT-related markers, Snail and CTGF, showed higher expression in HOMC-A4 cells than in the other two HOMC cells. Meanwhile, the three HOMC cells showed similar expression of the mesenchymal marker, vimentin. Thus, the expression of the major EMT-related genes correlated well with the morphological characteristics of the three HOMCs, indicating that several EMT-related transcription factors may be responsible for the morphological changes in HOMCs.

### Combination treatment of TGF-β and IL-1β induces morphological change of HOMC-D4 cells

Our findings suggest that IL-1β may be involved in the morphological changes of these cells. In malignant mesothelioma cells, especially those in which the Hippo pathway is inactivated, EMT-related factors, including Snail, are also known to be upregulated by TGF-β signaling, which enhances EMT [12]. In view of these aspects, we treated HOMC cells with TGF-β and IL-1β to examine changes in cell morphology. When each of these factors was administered individually to HOMC-D4 cells, no morphological changes were observed (Fig. 4B). However, the morphology of HOMC-D4 cells was significantly changed to a fibroblast-like morphology when both factors were applied (Fig. 4B, C). In line with this observation, the decrease in E-cadherin expression and the increase in Snail and CTGF expression were more pronounced when TGF-β and IL-1β were administered together than when each was administered alone (Fig. 4D). Meanwhile, once sarcomatoid morphology was achieved, TGF-β and IL-1β were removed and the cells were further cultured for 1 week, but they did not return to epithelioid morphology (Additional file 1: Figure S1). These results suggest that TGF-β and IL-1β alone may not be sufficient to induce EMT and that they work in concert to induce more potent and unidirectional EMT in mesothelial cells.

### Lack of tumor formation in HOMC-transplanted mice

We previously reported that *NF2* knockdown or forced *YAP1* or *TAZ* expression in HOMC cell lines resulted in tumorigenicity in subcutaneously transplanted immunodeficient mice, while no tumor formation was detected when merely transplanting HOMC cell lines [16, 22]. In this study, we subcutaneously transplanted HOMC cell lines at further passage numbers, but no tumorigenicity was observed (Table 3). These results suggest that these HOMC mesothelial cell lines could be useful for evaluating future candidate cancer-related genes in mesothelial cells.

**Table 3:** Tumor formation in mice transplanted with HOMC cells

Cell line	Injection	Tumor formation
HOMC-B1	Subcutaneous	0/6 (0%)
HOMC-D4	Subcutaneous	0/3(0%)
HOMC-A4	Subcutaneous	0/3 (0%)

## Discussion

Currently, only a few immortalized mesothelial cell lines are available from public or commercial sources and can be used for experiments. In this study, we report three novel immortalized mesothelial cell lines established from mesothelial cells of a patient with gastric cancer. For immortalization of cells, we transduced the HPV *E6*/*E7* and *hTERT* genes. We showed that the three cell lines mimicked three major histological subtypes of MM, expressed mesothelial cell markers, showed some differences in mRNA expression profiling, and one of these lines, the HOMC-D4 line, could be induced for cell morphological transition with TGF-β/IL-1β treatment.

The ratios of histological subtypes of MM are ~60–70% for epithelioid, 20% for biphasic, and 10–20% for sarcomatoid [23]. Thus, creating novel control cell lines for epithelioid and biphasic types is important for studying the differentiation of mesothelial cells. In this regard, immortalized mesothelial cells (MeT-5A), which have been widely used, were initially established with the introduction of SV40-LT, showing an epithelial phenotype.

Although MM tissues from patients exhibit different histological subtypes, the underlying mechanisms are still largely unknown. One of the mechanisms might be that mesothelial cells have a de novo propensity to undergo EMT relatively easily, which has been termed MMT [2]. In this regard, several inflammatory cytokines, such as IL-1β, IL-6, and TGF-β, are thought to be involved in the MMT of mesothelial cells, such as EMT of epithelial cells [1, 24, 25]. Thus, we tested TGF-β and IL-1β in HOMC-D4 cells with intermediate morphology and found that they exhibited MMT to the sarcoma type when both factors were added simultaneously but not individually. Regarding the expression levels of these growth factors and cytokines, a microarray study also showed significant differences in IL-1β and IL-6 expression between HOMC-A4 (sarcomatoid type) and HOMC-D4 (intermediate type).

These cell lines did not show tumorigenicity when transplanted into nude mice, as we previously reported [16] and confirmed here again (Table 3). In contrast, when transplanted subcutaneously in mice, three HOMCs with *NF2* knockdown formed a tumor mass, confirming that *NF2* is an important tumor suppressor gene in MM cells [16]. Furthermore, we also previously reported that exogenous expression of active YAP1 or TAZ mutants resulted in tumorigenic effects in all three HOMCs [16, 22]. These results strongly indicate that MM cell transformation is induced by immortalized mesothelial cells with specific gene transduction or knockdown, indicating that HOMC cells can be used to screen novel genes that are responsible for MM development.

Finally, since these cell lines originated from a single patient, almost all genetic backgrounds were uniform among these lines, although some chromosomal alterations were detected. Thus, we suspect that specific epigenetic modifications may account for these three histological types. Future comprehensive studies of these possible epigenetic alterations may identify new mechanisms of MMT in mesothelial cells.

## Conclusion

We successfully established three immortalized mesothelial cell lines with distinct morphologies. These immortalized HOMC cell lines could be very useful for detecting causes of MM cell transformation, as well as for drug screening and other experiments in future studies.

## Supplementary Information

**Additional file 1: Figure S1. **Irreversibility of induced sarcomatoid phenotype in HOMC-D4 cells. After induction of the sarcomatoid phenotype induced by TGF-β and IL-1β in HOMC-D4 cells, the cells were cultured without the factors for 7 days. The fibroblastic phenotype was not reversible (right).

## Data Availability

The microarray datasets analyzed during the current study are available in the Gene Expression Omnibus under accession number GSE128788.

## Abbreviations

MM: Malignant mesothelioma
EMT: Epithelial-to-mesenchymal transition
MMT: Mesothelial-to-mesenchymal transition
RTK: Receptor tyrosine kinase
