# Supplementary figures and images for: Three newly established immortalized mesothelial cell lines exhibit morphological phenotypes corresponding to malignant mesothelioma epithelioid, intermediate, and sarcomatoid types, respectively
Source: Cancer Cell Int. 2021 Oct 18;21:546. doi: 10.1186/s12935-021-02248-5 (PMC8525006; doi:10.1186/s12935-021-02248-5)

## Slide 1
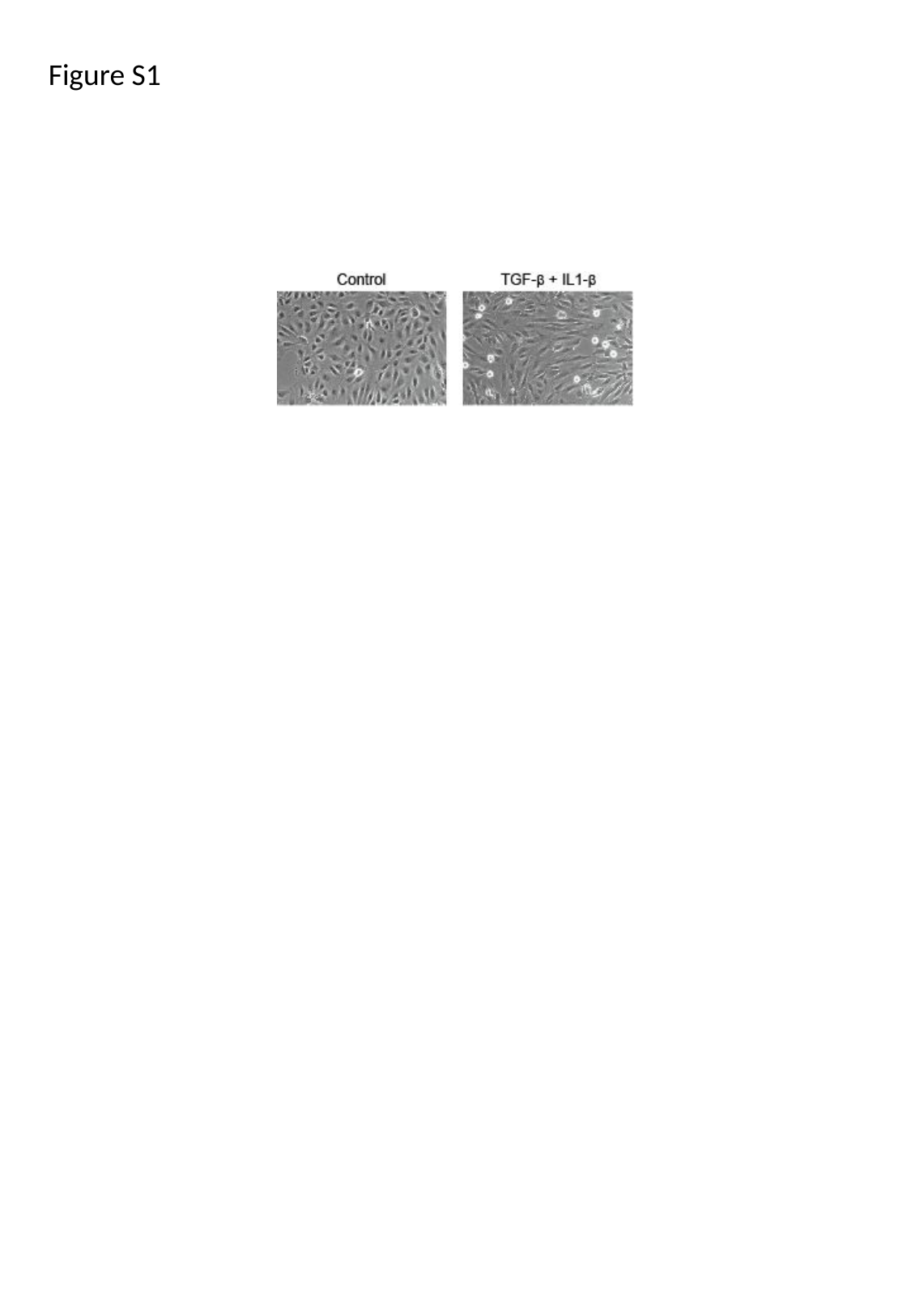

Figure S1

Supplement: Supplementary file 1 — Additional file 1: Figure S1. Irreversibility of induced sarcomatoid phenotype in HOMC-D4 cells. After induction of the sarcomatoid phenotype induced by TGF-β and IL-1β in HOMC-D4 cells, the cells were cultured without the factors for 7 days. The fibroblastic phenotype was not reversible (right). [file 12935_2021_2248_MOESM1_ESM.pptx]
